# Robot-Assisted Nephropexy

**DOI:** 10.1590/S1677-5538.IBJU.2017.0390

**Published:** 2018

**Authors:** Marcelo Langer Wroclawski, Guilherme Andrade Peixoto, Marcio Covas Moschovas, Arie Carneiro, Milton Borrelli, José Roberto Colombo

**Affiliations:** 1 Hospital Israelita Albert Einstein, São Paulo, SP, Brasil; 2 Departamento de Urologia, Faculdade de Medicina do ABC, Santo André, SP, Brasil

## Abstract

**Introduction:**

Renal ptosis is defined as the renal descent when there is a change from supine to orthostatic position, usually with a change of two vertebral bodies or more than five cm apart. Although rare, it is one of the causes of chronic flank pain or of upper abdomen. The typical patient of renal ptosis is young, female, thin, with complaint of pain when in an upright position (1, 2).

**Objective:**

Demonstrate a robot-assisted nephropexy technique in a young woman diagnosed with symptomatic renal ptosis on the right kidney, confirmed by imaging tests.

**Materials and Methods:**

A 29-year-old female patient with a history of chronic right-sided pain and palpable renal mobility on physical examination. The diagnosis of renal ptosis was confirmed by ultrasound imaging, excretory urography (Figure-1), and renal scintigraphy with 99mTc-DTPA (Figure-2). She was submitted to a robotic-assisted right nephropexy with a polypropylene mesh fixing the right kidney to the ipsilateral psoas muscle fascia.

**Result:**

We reported a 96-minute surgical time. The patient was discharged in the first postoperative day. At the one-month follow-up, there was an important improvement of the symptoms, with normality renal function and imaging tests describing adequate renal positioning.

**Conclusions:**

Robotic-assisted nephropexy is feasible and can be an excellent minimally invasive alternative technique for the proposed surgery. We reported a shorter hospital stay and a faster postoperative recovery compared with the opened procedure.

**Figure 1:**
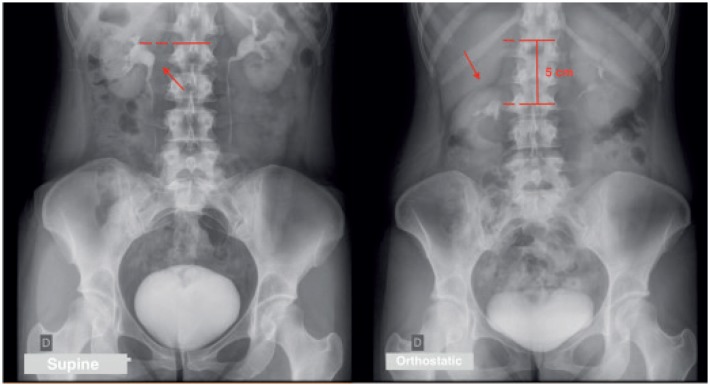
Excretory urography.

**Figure 2:**
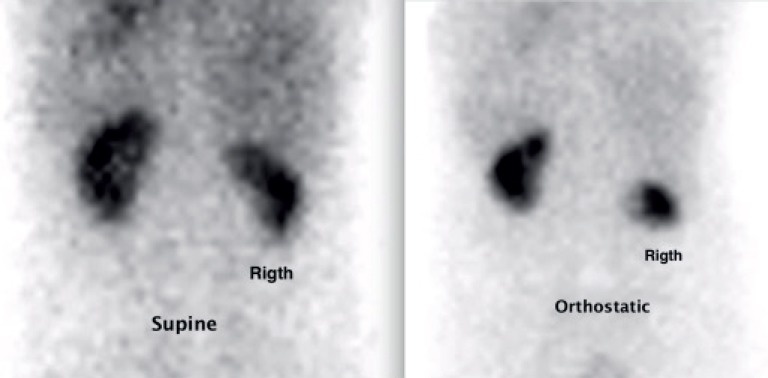
Renal scintigraphy.

## ARTICLE INFO

**Available at: http://www.intbrazjurol.com.br/video-section/20170390_wroclawski_et_al**

**Int Braz J Urol. 2018; 44 (Video #14): 1047-1048**

## References

[1] Kavoussi LR, Schwartz MJ, Gill IS (2011). Laparoscopic Surgery of the Kidney. Campbell-Walsh.

[2] Arnerlöv C, Söderström M, Öhberg L (2016). Mobile kidney pain provocation ultrasonography before surgery for symptomatic mobile kidney: A prospective study of 43 consecutive patients. Scand J Urol.

